# Impact of Potentially Antioxidant Probiotic Strains on Fermentation Quality and Antioxidant Status in Alfalfa Silage

**DOI:** 10.3390/antiox14040380

**Published:** 2025-03-24

**Authors:** Bokang Qi, Xinyu Cai, Wenkang Wang, Pengfei Ma, Xianjun Yuan, Xiang Tan

**Affiliations:** 1 Institute of Ensiling and Processing of Grass, College of Agro-Grassland Science, Nanjing Agricultural University, Nanjing 210095, China; 2023120018@stu.njau.edu.cn (B.Q.); 2022120016@stu.njau.edu.cn (X.C.); 2021820040@stu.edu.cn (W.W.); 2021820039@stu.edu.cn (P.M.); 2 Citrus Research Institute, Southwest University, Chongqing 400700, China

**Keywords:** antioxidant activity, alfalfa silage, lactic acid bacteria, fermentation quality

## Abstract

The study aimed to characterize the antioxidant properties of isolated lactic acid bacteria (LAB) and assess their impacts on fermentation quality and antioxidant status in alfalfa silage. Two LAB strains of *Lactiplantibacillus plantarum* XY15 and *Lactiplantibacillus plantarum* XY20 and a reference strain of *Pediococcus acidilactici* J17 were subjected to antioxidant property evaluation. This was followed by inoculation into alfalfa silage. The DPPH (2,2-diphenyl-1-picrylhydrazyl) and hydroxyl (OH) radical scavenging activities and the glutathione peroxidase (GSH-Px) activity of the cell-free supernatants of *L. plantarum* YX15 and *L. plantarum* YX20 were significantly (*p* < 0.05) higher than those of *P. acidilactici* J17. In all three strains, the superoxide dismutase (SOD) activity was higher in the cell-free supernatants than in the intracellular lysates. Among all three strains, *P. acidilactici* J17 showed the highest total antioxidant capacity (T-AOC) in the cell-free supernatant. Inoculating *L. plantarum* YX20 and *P. acidilactici* J17 increased lactic acid (LA) concentration and LAB counts, decreased dry matter (DM) loss, ammonia-N concentration, and pH, compared with control (CON) and *L. plantarum* XY15 inoculated alfalfa silages. After 1 d of ensiling, alfalfa silage inoculated with *L. plantarum* XY20 exhibited higher SOD activity than other silages. Inoculating *L. plantarum* XY20 and *P. acidilactici* J17 increased the DPPH free radical scavenging rates in alfalfa silage, compared with CON and *L. plantarum* XY15 inoculated 90 d-silages. Both *L. plantarum* YX15 and *L. plantarum* YX20 demonstrated a dual function of enhancing the lactic fermentation and improving the antioxidant status in alfalfa silage.

## 1. Introduction

Intensive livestock husbandry exposes animals to various stress factors. Notably, high-yield dairy cows frequently experience metabolic disorders, which, in turn, lead to severe oxidative stress. Under normal physiological conditions, the production and clearance of reactive oxygen species (ROS) are in a state of dynamic equilibrium. However, when animals are confronted with adverse challenges, this equilibrium will be disrupted, suppressing production performance and healthy growth [1,2]. To alleviate oxidative stress, various antioxidants have been incorporated into animal diets. This practice has been effective in improving animal survival rates and the shelf-life of animal products [3]. Probiotics have emerged as a potential alternative antioxidant source, primarily by enhancing the host’s tolerance to oxidative stress. Wang et al. [4] demonstrated that the *L. plantarum* ZLP001 retained over 95% of its original viable cell numbers after 240 min of incubation in 1.0 mmol/L hydrogen peroxide. Moreover, this strain exhibited a high scavenging ability against hydroxyl (OH), superoxide anion, and 2,2-diphenyl-1-picrylhydrazyl (DPPH) radicals. Wang et al. [5] reported that *L. plantarum* ZDY2013 had a significant impact on the dextran sodium sulfate-induced ulcerative colitis in mice by downregulating the expression of pro-inflammatory cytokines and upregulating the expression of antioxidant factors. Li et al. [6] reported that *Lactobacillus acidophilus* enhanced the antioxidant activity of *Rosa roxburghii* Tratt.

Alfalfa is a crucial forage and a fundamental component in the rations of dairy cattle. Its antioxidant properties can influence dietary antioxidant performance. However, alfalfa is challenging to ensile due to its low water-soluble carbohydrate (WSC) content but high buffering capacity. As a result, various inoculants are employed to enhance the fermentation quality of alfalfa silage [7]. However, there have been limited studies evaluating the impacts of inoculants on the antioxidant capacity of silages. Zhang et al. [8] demonstrated that the inoculation of *L. plantarum* 24-7, which has high-antioxidant capabilities, not only significantly inhibited the activity of molds and decreased the ammonia-N content but also increased the total antioxidant capacity (T-AOC), GSH-Px activity, and catalase (CAT) activity in alfalfa silages when compared with the control silages. Zhang et al. [9] reported that *P. acidilactici* J17 exhibited high-antioxidant capabilities and enhanced antioxidant status in alfalfa silages, which are indicated by higher T-AOC, GSH-Px, and CAT activities than in control. In our previous study [10], *L. plantarum* XY15 and *L. plantarum* XY20 were isolated from oat silage on the Tibetan plateau based on their high-antioxidant activity. Nevertheless, whether their antioxidant activity can be effectively exerted during the ensiling process remains unclear.

We hypothesized that the two LAB strains could not only enhance the fermentation quality but also augment the antioxidant capacity of alfalfa silage. Therefore, the study aimed to characterize the antioxidant properties of isolated lactic acid bacteria (LAB) and assess their impacts on fermentation quality and antioxidant status in alfalfa silage.

## 2. Materials and Methods

### 2.1. Antioxidant Properties of Three LAB Strains

Two LAB strains with potential antioxidant properties were isolated from the oat (*A. sativa*, cultivar LINGXIU) silage, which was naturally fermented without any additive. The two strains were identified as *L. plantarum* using 16S rRNA gene sequence and were designated XY15 and XY20, respectively. *P. acidilactici* J17 was isolated from perennial ryegrass silage by Zhang et al. [9], and it was identified as LAB with antioxidant activity in the previous study [11]. In the study, it served as a reference strain and was donated by the Probiotics and Biological Feed Research Centre of Lanzhou University. Based on the evaluation method proposed by Shimada et al. [12], both *L. plantarum* XY15 and *L. plantarum* XY20 possess high-antioxidant activity. These strains were stored as frozen (−80 °C) stocks in de Man, Rogosa, Sharpe (MRS) broth containing 20% (*v*/*v*) glycerol. Prior to utilization, the strains underwent at least three successive subcultures. In each subculture, a 1% (*v*/*v*) inoculum was added to MRS broth, which was then incubated at 37 °C for 18 h.

Both *L. plantarum* XY15 and *L. plantarum* XY20 were grown in MRS broth for 48 h at 37 °C under anaerobic conditions to produce stationary phase cultures. The supernatant of each strain was collected by centrifugation at 4 °C for 10 min at 8000× *g*. The bacterial cell pellets were washed twice with deionized water, followed by resuspending in deionized water. The intracellular lysate was prepared with slight modifications to the method described by Zhang et al. [13]. The cells were incubated with 1 mg/mL of lysozyme at 37 °C for 30 min, followed by ultrasonic disruption. The sonication was carried out in five 1-min intervals within an ice bath. Subsequently, the intracellular lysate was harvested by centrifugation at 8000× *g* for 10 min at 4 °C.

The DPPH and OH radical scavenging activities of LAB strains were determined following the method described by Ding et al. [14]. The T-AOC, GSH-Px, and SOD activities of different components of the three LAB strains were measured using assay kits (Nanjing Jiancheng Bioengineering Research Institute Co., Ltd., Nanjing, China) according to the instruction manual [11].

### 2.2. Alfalfa Silage Preparation

Alfalfa (*Medicago sativa* L.WL 324) was cultivated in the experimental field of Nanjing Agricultural University (32.04° N, 118.88° E, Nanjing, China). The first cutting alfalfa in each plot was harvested at the early bloom stage, and four experimental plots were utilized as replicates for each treatment. The harvested alfalfa was chopped into a theoretical length of 1–2 cm pieces with a forage chopper and then wilted to the dry matter (DM) content, reaching approximately 300 g/kg of fresh weight (FW). The forages from each plot were partitioned into twenty 500-g piles and randomly allocated to the following treatments: (1) distilled water (5 mL); untreated (CON); (2) *L. plantarum* XY15 to achieve 1 × 10^6^ cfu/g FW (IL); (3) *L. plantarum* XY20 to achieve 1 × 10^6^ cfu/g FW (IP); (4) *P. acidilactici* J17 to achieve 1 × 10^6^ cfu/g FW (J). Prior to application, microbial inoculants were cultured on MRS agar to verify viability, and appropriate amounts of the inoculants were used to achieve the desired application rate. Each inoculant was separately dissolved in 100 mL of deionized water to prepare a uniform suspension. A mini-sprayer was employed to apply 5 mL of the inoculant solution per pile, thoroughly mixing the strains with the forages. In the CON group, the same volume of sterile water was applied. Subsequently, all the treated samples were filled into polythene plastic bags (dimensions 350 mm × 450 mm) and vacuum-sealed strictly by using a vacuum sealer (DZD-400, Nanjing Omite Technology Co., Ltd., Nanjing, China). After that, the sealed bags were stored at room temperature (25 ± 2 °C).

### 2.3. Sampling and Analyses

Four silos for each treatment were randomly selected for sampling after 1, 5, 14, 45, and 90 d of ensiling, respectively. The fresh or ensiled alfalfa per silo was placed into a plastic container disinfected with ethanol and thoroughly mixed. Subsequently, it was divided into three subsamples.

One subsample (20 g) was homogenized in 180 mL of distilled water for 60 s by a juice extractor. The resulting mixture was then filtered through four layers of skim gauze and Whatman filter paper (pore size of 11 µm, Xinhua Co., Hangzhou, China). The silage pH was immediately detected with a glass electrode pH meter (Hanna pH 211, Hanna Instruments Italia Srl, Padova, Italy). Then, the filtrate was divided into three aliquots. One aliquot of the filtrate was acidified by using 50% H_2_SO_4_ (*w*/*w*) and centrifuged at 12,000× *g* for 10 min at 4 °C to collect the supernatant. The supernatant was used for the determination of organic acids (including LA, AcA, PA, and BA) and ethanol by high-performance liquid chromatograph (Agilent HPLC 1260, Agilent Technologies, Inc., Santa Clara, CA, USA). The second aliquot of the filtrate was subject to ammonia-N analysis using the phenol-sodium hypochlorite colorimetric method [15]. The third aliquot of the filtrate was used for radical scavenging activities and enzyme activity analyses, following the methods described above [11,14]. The 2,2′-azinobis (3-ethylbenzothiazoline-6-sulfonic acid) (ABTS) radical scavenging activities were analyzed according to the method used by Zhao et al. [16].

The second subsample (about 100 g) of both fresh alfalfa and silages was dried at 65 °C for 48 h to determine the DM content. After drying, the samples were ground using a grinder and then sieved through a 1 mm sieve. The WSC content in fresh alfalfa and silages was assessed using the colorimetric method described by Arthur Thomas [17]. The neutral detergent fiber (NDF) and acid detergent fiber (ADF) were determined using an Ankom200 Fiber Analyzer (ANKOM Technologies, Macedon, NY, USA) following the method of Van Soest et al. [18]. The flavonoid and total phenol contents were respectively measured using the Plant Flavonoids Content Assay and the Plant Total Phenol Content Assay Kits (Nanjing Jiancheng Bioengineering Research Institute Co., Ltd., Nanjing, China). The measurements were carried out in strict accordance with the instruction manuals of the respective kits.

The third subsample (about 10 g) of both fresh alfalfa and silage was mixed with 90 mL of sterile saline solution in a constant temperature (37 ± 2 °C) shaker for 20 min (120 rpm/min). Subsequently, the solution was filtered through four layers of sterile gauze. A 1 mL volume of filtrate was taken for microbial counting by 10-fold serial dilution. In total, 100 µL from an appropriate dilution was spread on agar plates. The count of LAB was determined by anaerobic culturing on MRS agar for 48 h at 37 °C. The count of yeast and molds was assessed on Potato Dextrose Agar (PDA) after 48 h of aerobic incubation at 30 °C. The microbial data were expressed as CFU and then transformed into logarithmic form based on the FW of the sample.

### 2.4. Statistical Analysis

Data regarding the chemical characteristics and microbial counts of fresh alfalfa were subjected to descriptive statistics using SPSS (SPSS 27.0, SPSS, Inc., Chicago, IL, USA).

Data on the antioxidant properties of three LAB strains, as well as fermentation quality and antioxidant properties of alfalfa silage, were analyzed using two-way analysis of variance (ANOVA) in SPSS based on a 4 × 5 factorial arrangement within a completely randomized design model. Interaction effects between factors were assessed, and post hoc multiple comparisons among the means were conducted using Tukey’s test, with statistical significance set at *p* < 0.05. Results are presented as means ± Standard Error of the Mean (SEM), and 95% confidence intervals (CIs) were calculated to assess the precision of the estimates.

## 3. Results

### 3.1. Antioxidant Properties of the Three LAB Strains

As presented in Table 1, a significant interaction (*p* < 0.05) between the media and the strains for the DPPH radical scavenging rates, OH radical scavenging rates, GSH-Px activity, and T-AOC was observed. The cell-free supernatants and intracellular extracts from *L. plantarum* YX15 exhibited significantly higher (*p* < 0.05) DPPH radical scavenging rates than those of *L. plantarum* YX20 and *P. acidilactici* J17. In contrast, the DPPH radical scavenging rates of bacterial cell suspension from *P. acidilactici* J17 were significantly higher (*p* < 0.05) than those of *L. plantarum* YX20 and *L. plantarum* YX15.

The intracellular extracts of *L. plantarum* YX20 exhibited the lowest (*p* < 0.05) OH radical scavenging rates than those of *L. plantarum* YX15 and *P. acidilactici* J17. In contrast, the OH radical scavenging rates of cell-free supernatants and bacterial cell suspension from *P. acidilactici* J17 were lower (*p* < 0.05) than those of *L. plantarum* YX15 and *L. plantarum* YX20.

The cell-free supernatant of the three strains had higher (*p* < 0.05) SOD activity than intracellular extracts. The GSH-Px activity of the cell-free supernatant of *L. plantarum* YX15 and *P. acidilactici* J17 was significantly (*p* < 0.05) higher than that of their intracellular extracts, whereas there was no difference for *L. plantarum* YX20. The T-AOC of the cell-free supernatant of *L. plantarum* YX20 and *P. acidilactici* J17 was significantly (*p* < 0.05) higher than that of their intracellular extracts; however, no difference was observed between cell-free supernatant and intracellular of *L. plantarum* YX15.

### 3.2. The Effect of Three LAB Strains on the Fermentation and Antioxidant Status of Alfalfa Silages

The chemical composition and microbial counts in fresh alfalfa are presented in Table 2. The fresh alfalfa was wilted to the DM concentration of 231 g/kg FW. The WSC, NDF, and ADF concentrations were 39.9 g/kg DM, 292 g/kg DM, and 238 g/kg DM, respectively. The counts of LAB and yeast and molds in alfalfa before ensiling were 8.04 log_10_ CFU/g FW and 6.78 log_10_ CFU/g FW, respectively.

The antioxidant activities in fresh alfalfa are presented in Table 2. The DPPH, OH, and ABTS free radical scavenging rates of fresh alfalfa were 18.0%, 71.0%, and 52.8%, respectively. The T-AOC, SOD activity, and GSH-Px activity of fresh alfalfa were 9.34 U/g FW, 91.2 U/g FW, and 503 U/g FW. The content of flavonoid and total phenol in alfalfa prior to ensiling was 3.8 mg/g DM and 0.025 mg/g DM, respectively.

As shown in Table 3, there was an interaction between inoculants and ensiling duration for the DM, WSC, and ammonia-N contents, LAB, and yeast counts. The DM content in CON, IL, and IP silages gradually decreased, while that in J silage remained stable over the 90 d of ensiling. The WSC concentration in all silages sharply decreased from 39.9 g/kg DM in fresh alfalfa to 27.8 g/kg DM after 1 d of ensiling, followed by a gradual decline until 90 d of ensiling. After 45 d of ensiling, the WSC concentration in IL silage was the highest (*p* < 0.05), while that in J was the lowest (*p* < 0.05) among all silages. After 14 d of ensiling, the ammonia-N concentration in CON increased to a higher (*p* < 0.05) level than other silages, while that in J silage remained the lowest (*p* < 0.05) until d 90.

The LAB counts in all silages remained stable during the initial 14 d of ensiling, followed by a sharp decline. After 45 d of ensiling, the IP and J silages exhibited higher (*p* < 0.05) LAB counts than CON and IL silages. The LAB counts in IL were significantly (*p* < 0.05) higher on d 45, while numerically (*p* > 0.05) higher than that in CON on d 90. Yeast counts in all silages decreased after 45 d of ensiling, and those in IL, IP, and J silages decreased below the detectable level on d 90.

As shown in Table 4, there was an interaction between inoculants and ensiling duration for pH, LA/AcA, LA, AcA, PA, and ethanol concentration. The pH in CON silage remained above 5.0 and the highest (*p* < 0.05) value among all silages over the entire ensiling process. The pH in IL and IP silages decreased below 5.0 after d 1 and remained stable during the initial 14 d of ensiling, followed by a slight increase. The pH in J silage remained stable from d 5 of ensiling. After 45 d of ensiling, the pH in J and IP silages was lower (*p* < 0.05) than that of IL silage. Although the CON silage had the lowest (*p* < 0.05) LA concentration during the first 5 d of ensiling, its LA concentration showed an increasing trend during the first 14 d of ensiling, followed by a slight decrease. The LA concentration in IL and IP silages increased and peaked on d 5 and d 14, respectively, followed by a slight decline. In contrast, the LA concentration in J silage gradually increased until d 90. The AcA concentration in all silages significantly (*p* < 0.05) increased over the ensiling process. After 45 d of ensiling, the AcA concentration in IL silage was significantly (*p* < 0.05) lower than that in other silages. The LA/AcA in all silages decreased as the ensiling process progressed. During the first 5 d of ensiling, the LA/AcA in IL and IP silages was higher (*p* < 0.05) than that in CON and J silages. However, the LA/AcA in J silage became significantly higher (*p* < 0.05) than that of CON and IL after 45 d of ensiling and remained the highest (*p* < 0.05) LA/AcA on d 90. The CON silage showed the highest (*p* < 0.05) PA concentration among all silages, except on d 45 of ensiling. The PA concentration in silages increased significantly from d 1 to 5 of ensiling. Subsequently, the PA concentration in IL significantly (*p* < 0.05) increased over the entire ensiling, while the PA concentration in IP and J significantly (*p* < 0.05) increased during 45 d of ensiling, followed by a gradual (*p* < 0.05) increase. The BA concentration in all silages was below the detectable level.

The CON silage showed the (*p* < 0.05) highest ethanol concentration over the ensiling processes. The ethanol concentration in IL silage increased and peaked on d 14, followed by a slight decline. The ethanol concentration in the J silage remained stable until d 45, followed by a slight decline (*p* < 0.05) until d 90.

As shown in Table 5, there was an interaction between inoculants and ensiling duration for DPPH, OH free radical scavenging rates. The DPPH free radical scavenging rates in IP and J silages were significantly (*p* < 0.05) higher than those of CON and IL after 90 d of ensiling. The IP and J silages had (*p* < 0.05) higher DPPH free radical scavenging rates on 90 d than those on 1 d, and the CON and IL remained stable over the ensiling. The CON silage had the (*p* < 0.05) lowest OH free radical scavenging rates among all silages on d 1, followed by a significant (*p* < 0.05) increase after 90 d of ensiling. The OH free radical scavenging rates in J silage significantly (*p* < 0.05) decreased after 90 d, while those in IL and IP remained stable over the ensiling. The ensiling duration significantly affected (*p* < 0.05) the ABTS free radical scavenging rates and the T-AOC. The silages on d 90 had higher (*p* < 0.05) ABTS free radical scavenging rates and T-AOC than those on d 1.

As shown in Table 5, there was an interaction between inoculants and ensiling duration for SOD activity. The IP silage exhibited a significantly (*p* < 0.05) higher SOD activity than CON, IL, and J silages on d 1. However, the SOD activity in CON was higher than that in IP and J silages after 90 d of ensiling. The SOD activity in CON significantly (*p* < 0.05) increased, while that in IP and J silages significantly decreased over the ensiling.

The ensiling duration significantly affected (*p* < 0.05) the GSH-Px activity, the flavonoid content, and the total phenol content. Regardless of treatments, the 90 d silages had higher (*p* < 0.05) flavonoid and total phenol contents but lower (*p* < 0.05) GSH-Px activity than the corresponding 1 d silages.

## 4. Discussion

### 4.1. Antioxidant Activities of L. plantarum XY15 and L. plantarum XY20

Based on the potential antioxidant activities exhibited by *L. plantarum* XY15 and *L. plantarum* XY20 in a previous study [10], these two strains were selected to evaluate their antioxidant properties during *in vitro* culture and ensiling. *P. acidilactici* J17 had been proven to possess high-antioxidant activity and was used as a reference strain in the study [9]. In intensive livestock husbandry, high-yield dairy cows are more vulnerable to oxidative stress because of their intense metabolic demands for both maintenance and production, resulting in metabolic and infectious diseases [19]. The LAB strains with antioxidant potential have been frequently reported to alleviate oxidative stress in animals. Ge et al. [20] found that *L. plantarum* NJAU-01 derived from traditional Chinese dry-cured ham could effectively alleviate oxidative stress in aging mice and regulate protein oxidation in fermented sausages. The antioxidant potential of LAB has been regarded as one of the criteria for evaluating probiotic functions [21]. Liu et al. [22] isolated *Lactobacillus paracasei* and *Lactobacillus martensii* from traditional fermented rice acid and found these two strains had high DPPH free radical scavenging activity. Kanno et al. [23] reported that *L. plantarum* 7FM10, isolated from the traditional Japanese food narezushi, exhibited DPPH and superoxide radical scavenging capabilities. In the study, the cell-free supernatant of *L. plantarum* XY20 demonstrated higher DPPH and OH free radicals scavenging activities than that of *P. acidilactici* J17. The cell-free supernatant and intracellular extracts of *L. plantarum* XY15 exhibited higher DPPH radical scavenging activity than *P. acidilactici* J17.

These results indicated that both *L. plantarum* XY20 and XY15 possessed antioxidant properties, which might be related to the origin of the two strains. Both strains were isolated from the harsh conditions of the Qinghai-Tibet Plateau, which is characterized by low temperature, low oxygen, and intense ultraviolet radiation. During the long evolution, the phyllosphere microorganisms gradually developed the antioxidant capacity to adapt to these harsh conditions. Liu et al. [24] also demonstrated that microorganisms can adapt to the extreme conditions in the glaciers of the Tibetan Plateau. For the three strains, the cell-free supernatants exhibited the highest DPPH radical scavenging rates, whereas the intracellular extracts demonstrated the greatest OH radical scavenging activities. This implies that the scavenging effect of DPPH radicals by the three strains depends on extracellularly secreted substances, whereas the scavenging effect of OH radicals requires intracellular substances. Liu et al. [25] indicated that *L. plantarum* NTU 102, isolated from homemade Korean-style cabbage pickles, had the capacity to produce exopolysaccharides. These exopolysaccharides exhibited inhibitory effects on conjugated dienes, DPPH-scavenging activity, reducing power, and metal-chelation activity. Lin and Yen [26] conducted an investigation into the OH radical scavenging ability of intracellular extracts derived from 19 LAB strains. They discovered that the majority of these extracts displayed strong scavenging activity.

Previous studies indicated that LAB could protect against oxidative stress by producing microbial antioxidant metabolites [27]. The SOD and GSH-Px are intracellular antioxidant enzymes that play crucial roles in defending against oxidative stress. The SOD mainly exists in the cytoplasm and mitochondria and catalyzes the conversion of superoxide anions into hydrogen peroxide. Subsequently, the generated hydrogen peroxide can be further decomposed into water and oxygen, thereby protecting cells from damage [28]. The GSH-Px can promote the decomposition of hydrogen peroxide. The T-AOC reflects the ability of the non-enzymatic antioxidant defense system. The SOD, GSH-Px, and T-AOC are important indicators for evaluating the in vitro antioxidant capacity of substances. In this study, the cell-free supernatant of *L. plantarum* XY15 and *L. plantarum* XY20 demonstrated higher GSH-Px activities but lower T-AOC than that of *P. acidilactici* J17. Simultaneously, the intracellular extracts of *L. plantarum* XY15 and *L. plantarum* XY20 exhibited higher GSH-Px activities and T-AOC levels than those of *P. acidilactici* J17. This indicates that the substances responsible for the antioxidant effects in these three strains are different. Yang et al. [29] isolated five LAB strains with strong T-AOC from the gastrointestinal tract of Hainan black goats. They discovered that the supernatants of these five strains had high SOD activity but weak GSH-Px enzyme activity. Abdel Tawab et al. [30] determined the activities of SOD and GSH-Px in the cellular extracts of four LAB strains isolated from Egyptian fermented food. They discovered that all four strains displayed high SOD enzyme activity, yet only two LAB strains, *Enterococcus lactis* IS05 and *L. plantarum* IS07, exhibited high GSH-Px activity. Yang et al. [31] reported that *L. paracasei* M11-4 isolated from fermented rice exhibited excellent antioxidant properties both in vitro and in vivo. The cell-free fermentation culture and the cell-content extracts of this strain demonstrated high T-AOC, as well as high activities of GSH-PX and SOD. For the three strains, the SOD activity in the cell-free supernatants was higher than that in the intracellular extracts. This finding might be attributed to cell autolysis, which enhanced the release of intracellular SOD into the surrounding medium, thereby increasing its detected activity in the supernatants [32].

### 4.2. The Effects of L. plantarum YX15 and L. plantarum YX20 on the Fermentation Quality of Alfalfa Silages

Alfalfa silage is an important protein source for livestock due to its good nutritional value, high palatability, and year-round availability. Silage with high antioxidant properties can offer antioxidant-rich diets for livestock, thereby mitigating various stresses encountered in livestock production. In the study, the three antioxidant LAB strains were inoculated to alfalfa to assess their impacts on fermentation quality and antioxidant status in silages.

The DM concentration in CON, IL, and IP silages gradually decreased. In contrast, the DM content in the J silage remained stable during ensiling. This indicates that the inoculation of *P. acidilactici* J17 mitigated the DM loss of alfalfa silage during the ensiling process. Sun et al. [33] also found that the inoculation of *Lactobacillus brevis* R33 reduced the DM loss of rice straw silage.

The IP and J silages showed higher LA concentration and lower pH (<4.70) than the CON silage over the 90 d of ensiling. This finding indicated that the inoculation of *L. plantarum* YX20 and *P. acidilactici* J17 effectively enhanced the LA production, thereby decreasing silage pH. In contrast, Zhang et al. [9] found that pH in alfalfa inoculated with commercial *L. plantarum* MTD-1 decreased to about 5.50 after 60 d of ensiling, regardless of DM contents. Driehuis et al. [34] reported that co-inoculation of *P. pentosaceus* and *L. plantarum* accelerated the decline in pH during the initial stage of ensiling. After 90 d of ensiling, the silages with the combined inoculation had a lower final pH but a higher lactic acid concentration than the uninoculated silages. Zhang et al. [11] inoculated two *P. acidilactici* strains to alfalfa and found both strains increased the LA concentration and reduced the pH and ammonia-N concentration as compared with the control. Sun et al. [35] also revealed that *P. acidilactici* could rapidly colonize during the early stage of oat silages and produce LA to inhibit undesirable microbes.

In the study, after 45 d of ensiling, the LA concentration in IL silage was the lowest among the inoculated silages, while that in J silage was the highest. This indicates that the J silage underwent more extensive fermentation than the IL silage. This finding was further corroborated by the lowest WSC concentration in J silage after 45 d of ensiling. Similarly, Li et al. [36] found that the alfalfa silage inoculated with *L. plantarum* 24-7 had the highest LA concentration but the lowest WSC concentration among four silages after 90 d of ensiling. They deduced that *L. plantarum* 24-7 could efficiently metabolize WSC into LA. In the study, the WSC concentration in CON silage significantly decreased after 5 d of ensiling; however, its LA concentration remained the lowest. This contradictory result might be attributed to the proliferation of undesirable bacteria. Yang et al. [37] found that the rapid decline in WSC in alfalfa silage was associated with the proliferation of *Hafnia* and the accumulation of BA during the initial 10 d of ensiling. Zhao et al. [38] revealed that the dominance of *Enterobacter* and *Garciella* in alfalfa silages was responsible for the low WSC concentration, along with the high pH and ammonia-N concentrations.

In the study, the inoculated silages maintained a lower ammonia-N concentration than the CON silage after 14 d of ensiling. Ammonia-N, one of the proteolysis products during ensiling, is a result of the synergistic effects of plant protease and clostridial fermentation. In the study, inoculation of LAB effectively promoted a decrease in pH, inhibiting the proliferation of undesirable bacteria. Zhang et al. [11] discovered that alfalfa silages inoculated with *L. plantarum* AS21, *L. plantarum* FM15, *P. acidilactici* 13-7, and *P. acidilactici* J17 had significantly lower ammonia-N concentrations than the control on d 60.

The higher LAB counts in IL, IP, and J silages than CON silages on d 45 were related to the inoculated LAB. After 90 d of ensiling, the undetectable yeast levels in the inoculated silages might be ascribed to acidic and anaerobic conditions. Liu et al. [39] also discovered that, throughout the ensiling process, the LAB-inoculated barley silage had a higher LAB count but lower numbers of yeasts and aerobic bacteria compared to the control.

Both *L. plantarum* YX15 and *L. plantarum* YX20 used in the study are facultative heterofermentative strains. According to Muck et al. [40], these strains can not only ferment hexoses into LA but also ferment pentoses into LA and AcA. *L. plantarum,* typically exhibits a homofermentative pathway when an excess of glucose is present. However, as Borch et al. [41] noted, under conditions of limited carbohydrates, it will switch to a heterofermentative pathway. In the study, *L. plantarum* YX15 and *L. plantarum* YX20 had more efficient lactate fermentation than that in CON and J during the first 5 d of ensiling. As WSC fermented, the inoculants of *L. plantarum* YX15 and *L. plantarum* YX20 gradually shifted to a heterofermentative pattern, resulting in the AcA accumulation.

Li et al. [36] reported that alfalfa silage inoculated with commercial *L. plantarum* MTD-1 had lower PA concentration than CON silages during the first 14 d of ensiling; however, the PA concentration gradually increased and peaked on d 90. In our study, the IL, IP, and J silages remained at a lower PA concentration than the CON silage during the first 14 d of ensiling. This phenomenon was attributed to the rapid and extensive LA fermentation of inoculated LAB, which effectively inhibited the metabolism of undesirable microorganisms. As the ensiling fermentation progressed, the PA concentration in the inoculated silages gradually increased to a level similar to that in the CON silage. This increase might be attributed to the conversion from LA to PA. Isipato et al. [42] reported that lactate could be converted to propionate through either direct reduction or the dicarboxylic acid pathway. During this conversion, acetate and CO_2_ are produced as by-products. Woolford [43] found that *Popionibacterium* species had been demonstrated to convert lactate into propionate in low pH silages. Moreover, these bacteria prioritize lactate over sugar as a substrate.

The inoculated silages had a lower ethanol concentration than the CON silage over the ensiling processes. This observation indicates that the inoculated LAB effectively inhibited the activity of undesirable microorganisms such as yeasts. Ethanol is mainly metabolized from glucose by yeasts and enterobacteria during ensiling fermentation. However, a rapid decline in pH can inhibit this process, as noted by Dong et al. [44]. Filya et al. [45] discovered that the inoculation of *L. plantarum* led to a decrease in the ethanol concentration of first-cut alfalfa silages after 35 d of ensiling.

### 4.3. The Effects of L. plantarum YX15 and L. plantarum YX20 on the Antioxidant Status of Alfalfa Silages

The bioactive compounds of flavonoids and phenols were detected in fresh alfalfa, contributing to the antioxidant properties of fresh alfalfa. Rafińska et al. [46] summarized that the combination of high biomass production and the high content of phenolic compounds and saponins renders alfalfa a good source of bioactive compounds.

Furthermore, dietary inclusion of high-antioxidant silage confers significant nutritional and physiological benefits to ruminants. Zhang et al. [47] reported that feeding dairy goats with alfalfa silage inoculated with Lactobacillus plantarum 24-7 increased antioxidant enzyme activities, immune function, and milk quality in lactating goats and upregulated antioxidant-related gene expression in the mammary gland. Li et al. [19] found that inoculation with ferulic acid esterase-producing *L. plantarum* A1 improved silage antioxidant capacity, nutrient digestibility, and rumen fermentation because ferulic acid exhibited antioxidant properties. They also demonstrated that feeding dairy goats with these silages enhanced antioxidant and immune responses, leading to increased milk fat and protein content. Tian et al. [48] showed that feeding lactating goats with anthocyanin-rich purple corn stalk (PSS) silage increased milk lactose content and enhanced antioxidant enzyme levels in plasma and milk. It is predicted that alfalfa silages inoculated with potential high-antioxidant LAB might benefit animals.

The DPPH, OH, and ABTS free radical scavenging rates are widely used to determine the in vitro antioxidant capacity of a substance. The DPPH free-radical scavenging rates of the IP and J silages were comparable to those of the CON and IL silages on d 1. However, the DPPH free-radical scavenging rates in the IP and J silages had increased markedly and were higher than those in the CON and IL silages on d 90. This finding is consistent with the higher DPPH free radical scavenging rates observed in the bacterial cell suspension of *L. plantarum* YX20 and *P. acidilactici* J17 than in *L. plantarum* YX15. It indicates that the inoculation of *L. plantarum* YX20 and *P. acidilactici* J17 has enhanced the DPPH free radical scavenging capacity in alfalfa silage. Kachouri et al. [49] reported that using *L. plantarum* LAB 1 increased the antioxidant activity by 24% during storage of olive fruits.

After 90 d of ensiling, the OH free radical scavenging rates in CON silage significantly increased. This increase might be attributed to the rise of SOD activity. In contrast, the decline in OH free radical scavenging rates of J silage was associated with the decrease in GSH-Px activity. Both GSH-Px and SOD are components of the multicomponent oxidative system. This system can neutralize the ROS, including free oxygen radicals generated during metabolic processes [14,50]. The inoculated silages had higher OH free radical scavenging rates than CON on d 1, indicating that the inoculants could enhance the OH free radical scavenging rates in alfalfa silages.

The increase in the flavonoid and total phenol contents in silages during ensiling might be attributed to microbial metabolism and harsh acidic conditions. These factors may facilitate the release of bioactive compounds or the enzymatic metabolism of antioxidant substances into more active small molecules, thereby enhancing the response of the antioxidant system [51]. Liu et al. [52] also reported that plant-derived polyphenols could effectively scavenge reactive oxygen species in organisms, transfer electrons to free radicals to neutralize them, and improve the expression of antioxidant enzymes. Dong et al. [53] reported that fermentation using two commercial LAB strains increased the concentration of total phenolic, total flavonoids, and betaine in Chinese wolfberry, enhancing its antioxidant activity. Isas et al. [54] also found that pomegranate juice exhibited an increase in total phenolic concentration and antioxidant activity during fermentation with two *L. paraplantarum* strains. In the present study, the 90 d silages had higher ABTS free radical scavenging rates, flavonoid content, and total phenol content than the 1 d silages. These enhancements are likely attributed to the metabolic activities of LAB, particularly their ability to hydrolyze phenolic glycosides and generate bioactive compounds during fermentation.

After 90 d of ensiling, the CON and IL silages had higher SOD activity than IP and J silages. This phenomenon could be ascribed to the lower pH levels in the IP and J silages than in the CON and IL silages. The lower pH in the IP and J silages was likely to inhibit the SOD activity in the silage. Mohd Zin et al. [55] determined the antioxidative activities of *Morinda citrifolia* L. leaves extracted at different pH (pH 3 to pH 9) and found that SOD enzymes activity increased as the pH increased from 4.0 to 7.0. Li et al. [36] found that the SOD activity in alfalfa silage inoculated with *L. plantarum* A1 and *L. plantarum* 24-7 most rapidly decreased among all silages during the initial 7 d of ensiling. Moreover, all the silages reached their lowest SOD activity values on d 90. Meanwhile, the IP silages had lower SOD activity but higher T-AOC activity on d 90 than d 1. This might be attributed to the presence of Mn^2+^, which has been demonstrated to exert antioxidant effects through mimicking SOD activity in conditions of enzymatic deficiency [56]. Archibald and Duong [57] reported that Lactobacillus plantarum accumulates intracellular Mn^2+^ up to 30 mM during growth.

GSH-Px catalyzes the reduction of hydrogen peroxide (H_2_O_2_) to water (H_2_O) using reduced GSH as a substrate, generating oxidized glutathione, which lacks antioxidant capacity. Oxidized GSH is regenerated by Glutathione Reductase into its reduced form, which serves as a substrate in the continuous elimination of reactive oxygen species [58]. In this study, the 90 d silages had lower GSH-Px activity than the corresponding 1 d silages. This finding is in agreement with the results of Li et al. [36], who found that the GSH-Px activity in alfalfa silage inoculated with *L. plantarum* A1 or the control both rapidly increased during the initial 7 d of ensiling; however, it decreased after 90 d of ensiling. This might be attributed to the inactivation and degradation of enzymes in acidic conditions and the lack of GSH produced by microorganisms. This finding was further corroborated by a significant reduction in microbial counts and harsh acidic conditions during ensiling.

## 5. Conclusions

The *L. plantarum* YX15 and *L. plantarum* YX20, which were isolated from oat silage from the Tibetan plateau, demonstrated antioxidant capacity. This was indicated by high DPPH and OH radical scavenging activities, as well as SOD and GSH-Px activity detected in the cell-free supernatants of the two strains. Inoculating *L. plantarum* YX20 and *P. acidilactici* J17 in alfalfa enhanced LA fermentation and suppressed undesirable fermentation, which are indicated by a lower ammonia-N and ethanol concentration compared with the control. Inoculating *L. plantarum* YX20 and *P. acidilactici* J17 increased the DPPH free radical scavenging rates in alfalfa silage compared with CON and *L. plantarum* XY15 inoculated 90d-silages.

## Figures and Tables

**Table 1:** The free radical scavenging activities and antioxidant properties of different components of three LAB strains.

Items	Media	Strains			Means	SEM	*p*-Value		
		YX15	YX20	J17			MED	S	MED × S
DPPH scavenging rates (%)	CS	89.0 ^Aa^	88.0 ^Ab^	84.8 ^Ac^	87.3	4.41	<0.001	<0.001	<0.001
	IE	84.4 ^Ba^	79.1 ^Bc^	81.6 ^Bb^	81.7				
	BS	13.2 ^Cc^	63.1 ^Cb^	70.0 ^Ca^	48.8				
OH scavenging rates (%)	CS	25.9 ^Bab^	31.7 ^Ba^	23.8 ^Bb^	27.1	27.71	<0.001	0.004	<0.001
	IE	47.9 ^Aa^	41.4 ^Ab^	48.5 ^Aa^	45.9				
	BS	12.5 ^Ca^	14.4 ^Ca^	3.37 ^Cb^	10.1				
SOD activity (U/mL)	CS	23.4	23.4	23.4	23.4 ^A^	15.701	<0.001	0.278	0.290
	IE	8.74	7.73	7.46	7.97 ^B^				
	BS	—	—	—	—				
GSH-Px activity (U/mL)	CS	115 ^Aa^	94.3 ^ab^	57.4 ^Ab^	89.0	73.52	<0.001	<0.001	0.045
	IE	59.4 ^Bab^	78.0 ^a^	36.9 ^Bb^	58.1				
	BS	—	—	—	—				
T-AOC (U/mL)	CS	0.141 ^c^	0.264 ^Ab^	0.333 ^Aa^	0.246	0.17955	<0.001	<0.001	<0.001
	IE	0.117 ^a^	0.124 ^Ba^	0.0971 ^Bb^	0.113				
	BS	—	—	—	—				

^a–c^ Means in the same row with unlike superscript differ (*p* < 0.05), ^A–C^ Means in the same column with unlike superscript differ (*p* < 0.05). DPPH, 2,2-diphenyl-1-picrylhydrazyl; OH, hydroxyl; SOD, superoxide dismutase; GSH-Px, glutathione peroxidase. T-AOC, total antioxidant capacity; YX15, *L. plantarum*-YX15; YX20, *L. plantarum*-YX20; J17, *P. acidilactici*-J17. CS, Cell-free supernatant; IE, Intracellular extracts; BS, Bacterial cell suspension. SEM, standard error of the mean. MED, media; S, strains; MED × S, interaction between medium and strains.

**Table 2:** Chemical compositions, microbial population, and antioxidant activities of fresh alfalfa before ensiling.

Items	Mean	SD
Dry matter (g/kg FW)	231	1.2
Water-soluble carbohydrates (g/kg DM)	39.9	4.24
Neutral detergent fiber (g/kg DM)	292	0.9
Acid detergent fiber (g/kg DM)	238	0.3
Lactic acid bacteria (log_10_ CFU/g FW)	8.04	0.046
Yeasts (log_10_ CFU/g FW)	6.78	0.240
DPPH free radical scavenging rates (%)	18.0	1.39
OH free radical scavenging rates (%)	71.0	1.73
ABTS free radical scavenging rates (%)	52.8	1.91
T-AOC ability (U/mL)	9.34	2.871
SOD activity (U/mL)	91.2	33.67
GSH-Px activity (U/mL)	503	3.2
Flavonoid content (mg/g DM)	3.80	1.26
Total phenol content (mg/g DM)	0.0250	0.01183

FW, fresh weight; DM, dry matter; CFU, colony-forming units. SD, standard deviation. DPPH, 2,2-diphenyl-1-picrylhydrazyl; OH, hydroxyl; ABTS,2,2′-azino-bis(3-ethylbenzothiazoline-6-sulfonic acid); T-AOC, total antioxidant capacity; SOD, superoxide dismutase; GSH-Px, glutathione peroxidase.

**Table 3:** Effects of inoculating LAB on chemical compositions and microbial population of alfalfa silage.

Items	Treatments	Ensiling Days					SEM	*p*-Value		
		1	5	14	45	90		INO	D	INO × D
Dry matter (g/kg FW)	CON	240 ^a^	219 ^Bab^	214 ^Bab^	205 ^Cb^	218 ^Bab^	1.4	<0.01	<0.01	<0.01
	IL	224 ^ab^	227 ^ABa^	223 ^ABab^	212 ^Cb^	214 ^Bb^				
	IP	240 ^a^	230 ^Aab^	225 ^ABab^	224 ^Bab^	219 ^Bb^				
	J	231 ^b^	231 ^Ab^	233 ^Aab^	236 ^Aab^	239 ^Aa^				
Water-soluble carbohydrates (g/kg DM)	CON	27.1 ^a^	23.8 ^b^	18.2 ^c^	16.4 ^ABc^	18.1 ^Bc^	0.50	<0.01	<0.01	<0.01
	IL	28.2 ^a^	23.3 ^b^	17.0 ^c^	18.8 ^Ac^	21.5 ^Ab^				
	IP	28.1 ^a^	22.1 ^b^	17.8 ^cd^	16.4 ^ABd^	19.9 ^ABbc^				
	J	27.7 ^a^	20.8 ^b^	17.4 ^bc^	15.1 ^Bc^	14.6 ^Cc^				
Ammonia nitrogen (%DM)	CON	1.24 ^d^	3.45 ^Ac^	5.66 ^Ab^	7.40 ^Aa^	8.02 ^Aa^	0.224	<0.01	<0.01	<0.01
	IL	1.12 ^e^	2.28 ^Bd^	3.75 ^Bc^	5.24 ^Bb^	6.02 ^Ba^				
	IP	0.90 ^d^	2.55 ^Bc^	3.54 ^BCb^	4.55 ^Ca^	4.85 ^Ca^				
	J	1.02 ^d^	3.09 ^Aab^	2.88 ^Cc^	3.29 ^Db^	4.40 ^Ca^				
Lactic acid bacteria (log_10_ CFU/g FW)	CON	8.44 ^a^	8.53 ^a^	8.10 ^a^	5.66 ^Cb^	5.98 ^Bb^	0.125	<0.01	<0.01	<0.01
	IL	8.66 ^a^	8.62 ^a^	8.17 ^a^	6.13 ^Bb^	6.16 ^Bb^				
	IP	8.57 ^a^	8.68 ^a^	8.25 ^a^	6.70 ^Ab^	6.59 ^Ab^				
	J	8.49 ^a^	8.79 ^a^	8.49 ^a^	6.94 ^Ab^	6.57 ^Ab^				
Yeasts (log_10_ CFU/g FW)	CON	7.48 ^Aa^	7.27 ^a^	7.01 ^a^	5.92 ^b^	5.27 ^b^	0.284	<0.01	<0.01	<0.01
	IL	7.15 ^Bb^	7.61 ^a^	7.08 ^b^	6.15 ^c^	<2				
	IP	7.35 ^ABa^	7.49 ^a^	6.94 ^b^	6.41 ^c^	<2				
	J	7.28 ^ABa^	7.40 ^a^	6.86 ^a^	5.75 ^b^	<2				

^a–e^ Means in the same row with unlike superscript differ (*p* < 0.05), ^A–D^ Means in the same column with unlike superscript differ (*p* < 0.05). FW, fresh weight; DM, dry matter. SEM, standard error of the mean. INO, inoculants; D, ensiling days; INO × D, interaction between inoculants and ensiling days. CON, control; IL, *L. plantarum*-YX15 at 1 × 10^6^ CFU/g FW; IP, *L*. *plantarum*-YX20 at 1 × 10^6^ CFU/g FW; J, *P. acidilactici*-J17 at 1 × 10^6^ CFU/g FW.

**Table 4:** Effects of inoculating LAB on pH value and fermented products of alfalfa silage.

Items	Treatments	Ensiling Days					SEM	*p*-Value		
		1	5	14	45	90		INO	D	INO × D
pH	CON	5.40 ^Aa^	5.52 ^Aa^	5.53 ^Aa^	5.36 ^Aab^	5.08 ^Ab^	0.039	<0.01	<0.01	<0.01
	IL	4.56 ^Cc^	4.64 ^Bc^	4.76 ^Bbc^	5.15 ^Ba^	4.99 ^Bab^				
	IP	4.57 ^Cbc^	4.51 ^Bc^	4.61 ^Bbc^	4.85 ^Ca^	4.70 ^Cb^				
	J	4.91 ^Ba^	4.68 ^Bb^	4.61 ^Bb^	4.64 ^Db^	4.56 ^Db^				
Lactic acid (g/kg DM)	CON	20.2 ^Dbc^	36.7 ^Ca^	39.9 ^Ba^	16.5 ^Cc^	25.7 ^Cb^	1.43	<0.01	<0.01	<0.01
	IL	48.1 ^Aa^	51.6 ^Aa^	43.9 ^Ba^	25.4 ^Cb^	24.6 ^Cb^				
	IP	39.1 ^Bb^	53.7 ^Aa^	53.8 ^Aa^	42.6 ^Bab^	41.7 ^Bab^				
	J	32.6 ^Cc^	45.2 ^Bb^	47.5 ^ABb^	58.5 ^Aa^	55.5 ^Aa^				
Acetic acid (g/kg DM)	CON	10.1 ^Ad^	21.9 ^Ac^	22.5 ^c^	28.0 ^Bb^	39.3 ^Aa^	1.07	<0.01	<0.01	<0.01
	IL	9.44 ^Ad^	17.8 ^Bc^	22.0 ^b^	21.1 ^Cb^	31.0 ^Ba^				
	IP	7.94 ^Bd^	17.1 ^Bc^	24.8 ^b^	26.4 ^Bb^	38.1 ^Aa^				
	J	10.2 ^Ad^	21.2 ^Ac^	21.8 ^c^	33.3 ^Ab^	38.7 ^Aa^				
Propionic acid (g/kg DM)	CON	3.58 ^Ad^	6.17 ^Ac^	7.10 ^Abc^	8.09 ^b^	9.44 ^Aa^	0.281	<0.01	<0.01	<0.01
	IL	2.33 ^Be^	4.24 ^Bd^	6.05 ^Bc^	7.64 ^b^	10.2 ^Aa^				
	IP	2.31 ^Bc^	3.29 ^Cc^	6.30 ^Bb^	8.66 ^a^	8.93 ^ABa^				
	J	2.66 ^Bc^	4.06 ^BCb^	4.48 ^Cb^	7.78 ^a^	7.59 ^Ba^				
Lactic/acetic acid	CON	1.99 ^Ca^	1.68 ^Bb^	1.77 ^ab^	0.591 ^Cc^	0.653 ^Cc^	0.1369	<0.01	<0.01	<0.01
	IL	5.11 ^Aa^	2.92 ^Ab^	2.00 ^c^	1.22 ^Bd^	0.793 ^Cd^				
	IP	4.91 ^Aa^	3.16 ^Ab^	2.19 ^c^	1.61 ^ABd^	1.09 ^Be^				
	J	3.22 ^Ba^	2.13 ^Bb^	2.18 ^b^	1.77 ^Abc^	1.43 ^Ac^				
Ethanol (g/kg DM)	CON	5.41 ^Ac^	11.7 ^Aa^	10.5 ^Aab^	9.42 ^Ab^	7.04 ^Ac^	0.277	<0.01	<0.01	<0.01
	IL	3.60 ^Cc^	4.81 ^Bb^	6.42 ^Ba^	5.01 ^Bb^	5.44 ^Bb^				
	IP	2.88 ^Db^	3.92 ^Cab^	4.62 ^Ba^	3.34 ^Bab^	4.02 ^Cab^				
	J	4.51 ^Ba^	4.25 ^BCa^	4.68 ^Ba^	4.35 ^Ba^	3.38 ^Cb^				

^a–e^ Means in the same row with unlike superscript differ (*p* < 0.05), ^A–D^ Means in the same column with unlike superscript differ (*p* < 0.05). DM, dry matter. SEM, standard error of the mean. INO, inoculants; D, ensiling days; INO × D, interaction between inoculants and ensiling days. CON, control; IL, *L. plantarum*-YX15 at 1 × 10^6^ CFU/g FW; IP, *L*. *plantarum*-YX20 at 1 × 10^6^ CFU/g FW; J, *P. acidilactici*-J17 at 1 × 10^6^ CFU/g FW.

**Table 5:** Effects of inoculating 3 LAB on free radical scavenging rates, antioxidant capacity, and antioxidant contents of alfalfa silage within each ensiled day.

Items	Ensiling Days	Treatments				Mean	SEM	*p*-Value		
		CON	IL	IP	J			INO	D	INO × D
DPPH free radical scavenging rates (%)	Day1	18.0	19.2	17.0 ^B^	15.8 ^B^	17.5	3.85	<0.001	<0.001	<0.001
	Day90	19.8 ^b^	20.1 ^b^	69.0 ^Aa^	71.8 ^Aa^	45.2				
	Mean	18.9	19.7	43.0	43.8					
OH free radical scavenging rates (%)	Day1	64.6 ^Bb^	72.1 ^a^	71.0 ^ab^	77.8 ^Aa^	71.4	0.99	0.067	0.201	<0.001
	Day90	73.9 ^A^	69.9	64.7	69.3 ^B^	69.4				
	Mean	69.3	71.0	67.8	73.5					
ABTS free radical scavenging rates (%)	Day1	75.6	64.4	47.1	66.2	63.3 ^B^	3.01	0.052	<0.001	0.336
	D90	87.6	87.3	79.2	77.8	83.0 ^A^				
	Mean	81.6	75.9	63.1	72.0					
T-AOC ability (U/mL)	Day1	9.68	11.7	10.5	7.39	9.80 ^B^	0.82	0.543	<0.001	0.548
	Day90	17.3	17.1	16.1	16.8	16.8 ^A^				
	Mean	13.5	14.4	13.3	12.1					
SOD activity (U/mL)	Day1	12.5 ^Bb^	8.33 ^b^	20.0 ^Aa^	12.2 ^Ab^	13.3	1.47	<0.001	0.705	<0.001
	D90	25.6 ^Aa^	14.2 ^ab^	9.85 ^Bbc^	0.781 ^Bcd^	12.6				
	Mean	19.0	11.3	14.9	6.48					
GSH-Px activity (U/mL)	Day1	56.5	54.4	55.8	56.6	55.8 ^A^	0.59	0.697	<0.001	0.414
	Day90	52.3	53.0	49.9	51.6	51.7 ^B^				
	Mean	54.4	53.7	52.8	54.1					
Flavonoid content (mg/g DM)	Day1	3.68	4.89	5.07	5.03	4.67 ^B^	0.337	0.663	0.003	0.533
	Day90	6.84	6.29	5.92	7.54	6.65 ^A^				
	Mean	5.26	5.59	5.50	6.29					
Total phenol content (mg/g DM)	Day1	0.0142	0.0243	0.0235	0.0196	0.0204 ^B^	0.00219	0.328	0.009	0.409
	Day90	0.0350	0.0339	0.0359	0.0213	0.0315 ^A^				
	Mean	0.0246	0.0291	0.0297	0.0204					

^a–d^ Means in the same row with unlike superscript differ (*p* < 0.05), ^A,B^ Means in the same column with unlike superscript differ (*p* < 0.05). DM, dry matter; DPPH, 2,2-diphenyl-1-picrylhydrazyl; OH, hydroxyl; ABTS,2,2′-azino-bis(3-ethylbenzothiazoline-6-sulfonic acid); T-AOC, total antioxidant capacity; SOD, superoxide dismutase; GSH-Px, glutathione peroxidase. SEM, standard error of the mean. INO, inoculants; D, ensiling days; INO × D, interaction between inoculants and ensiling days. CON, control; IL, *L. plantarum*-YX15 at 1 × 10^6^ CFU/g FW; IP, *L*. *plantarum*-YX20 at 1 × 10^6^ CFU/g FW; J, *P. acidilactici*-J17 at 1 × 10^6^ CFU/g FW.

## Data Availability

Datasets used or analyzed in this study are available by reasonable request from the corresponding author.

## Abbreviations

The following abbreviations are used in this manuscript:

LAB	lactic acid bacteria
DPPH	2,2-diphenyl-1-picrylhydrazyl
OH	hydroxyl
SOD	superoxide dismutase
GSH-Px	glutathione peroxidase
T-AOC	total antioxidant capacity
CON	control
PA	propionic acid
ROS	reactive oxygen species
WSC	water-soluble carbohydrate
BA	butyric acid
LA	lactate
AcA	acetate
CAT	catalase
MRS	de Man, Rogosa and Sharpe medium
DM	Dry matter
FW	fresh weight
ABTS	2,2′-azinobis (3-ethylbenzothiazoline-6-sulfonic acid)
NDF	neutral detergent fiber
ADF	acid detergent fiber
rpm	revolutions per minute
PDA	Potato Dextrose Agar
CFU	colony-forming units
INO	inoculants
LA/AcA	The raito of lactate to acetate

## References

[1] Tafuri S., Cocchia N., Landolfi F., Iorio E.L., Ciani F. (2016). Redoxomics and Oxidative Stress: From the Basic Research to the Clinical Practice.

[2] Mirowska-Guzel D., Balkowiec-Iskra E. (2014). The Role of Monoamine Oxidase in Humans and Its Metabolism. Psychiatr. Ann..

[3] Chew B.P. (1996). Importance of antioxidant vitamins in immunity and health in animals. Anim. Feed Sci. Technol..

[4] Wang J., Ji H.F., Wang S.X., Zhang D.Y., Liu H., Shan D.C., Wang Y.M. (2012). *Lactobacillus plantarum* ZLP001: In vitro Assessment of Antioxidant Capacity and Effect on Growth Performance and Antioxidant Status in Weaning Piglets. Asian-Austral. J. Anim..

[5] Wang Y., Guo Y., Chen H., Wei H., Wan C. (2018). Potential of *Lactobacillus plantarum* ZDY2013 and Bifidobacterium bifidum WBIN03 in relieving colitis by gut microbiota, immune, and anti-oxidative stress. Can. J. Microbiol..

[6] Li B., Zhang T., Dai Y., Jiang G., Peng Y., Wang J., Song Y., Ding Z. (2023). Effects of probiotics on antioxidant activity, flavor compounds and sensory evaluation of *Rosa roxburghii* Tratt. LWT.

[7] Jaurena G., Pichard G. (2001). Contribution of storage and structural polysaccharides to the fermentation process and nutritive value of lucerne ensiled alone or mixed with cereal grains. Anim. Feed Sci. Technol..

[8] Zhang Y.X., Ke W.C., Vyas D., Adesogan A.T., Franco M., Li F.H., Bai J., Guo X.S. (2021). Antioxidant status, chemical composition and fermentation profile of alfalfa silage ensiled at two dry matter contents with a novel *Lactobacillus plantarum* strain with high-antioxidant activity. Anim. Feed Sci. Technol..

[9] Zhang Y.X., Ke W.C., Bai J., Li F.H., Xu D.M., Ding Z.T., Guo X.S. (2020). The effect of *Pediococcus acidilactici* J17 with high-antioxidant activity on antioxidant, α-tocopherol, β-carotene, fatty acids, and fermentation profiles of alfalfa silage ensiled at two different dry matter contents. Anim. Feed Sci. Technol..

[10] Yang X. (2022). Effects of Elevation Gradient on Phyllosphere Microbes and Fermentation Characteristics of *Kobresia pygmaea* and *Avena sativa* L.. Master’s Thesis.

[11] Zhang X., Guo X.S., Li F.H., Usman S., Zhang Y.X., Ding Z.T. (2022). Antioxidant, flavonoid, α-tocopherol, β-carotene, fatty acids, and fermentation profiles of alfalfa silage inoculated with novel *Lactiplantibacillus Plantarum* and *Pediococcus acidilactici* strains with high-antioxidant activity. Anim. Feed Sci. Technol..

[12] Shimada K., Fujikawa K., Yahara K., Nakamura T. (1992). Antioxidative properties of xanthan on the autoxidation of soybean oil in cyclodextrin emulsion. J. Agric. Food Chem..

[13] Zhang S., Liu L., Su Y., Li H., Sun Q., Liang X., Lv J. (2011). Antioxidative activity of lactic acid bacteria in yogurt. Afr. J. Microbiol. Res..

[14] Ding W., Wang L., Zhang J., Ke W., Zhou J., Zhu J., Guo X., Long R. (2017). Characterization of antioxidant properties of lactic acid bacteria isolated from spontaneously fermented yak milk in the Tibetan Plateau. J. Funct. Foods.

[15] Weatherburn M. (1967). Phenol-hypochlorite reaction for determination of ammonia. Anal. Chem..

[16] Zhao H., Fan W., Dong J., Lu J., Chen J., Shan L., Lin Y., Kong W. (2008). Evaluation of antioxidant activities and total phenolic contents of typical malting barley varieties. Food Chem..

[17] Arthur Thomas T. (1977). An automated procedure for the determination of soluble carbohydrates in herbage. J. Sci. Food Agric..

[18] Van Soest P.J., Robertson J.B., Lewis B.A. (1991). Methods for dietary fiber, neutral detergent fiber, and nonstarch polysaccharides in relation to animal nutrition. J. Dairy Sci..

[19] Li F., Zhang B., Zhang Y., Zhang X., Usman S., Ding Z., Hao L., Guo X. (2022). Probiotic effect of ferulic acid esterase-producing *Lactobacillus plantarum* inoculated alfalfa silage on digestion, antioxidant, and immunity status of lactating dairy goats. Anim. Nutr..

[20] Ge Q., Chen S., Liu R., Chen L., Yang B., Yu H., Wu M., Zhang W., Zhou G. (2019). Effects of *Lactobacillus plantarum* NJAU-01 on the protein oxidation of fermented sausage. Food Chem..

[21] Yu X., Li S., Yang D., Qiu L., Wu Y., Wang D., Shah N.P., Xu F., Wei H. (2016). A novel strain of Lactobacillus mucosae isolated from a Gaotian villager improves in vitro and in vivo antioxidant as well as biological properties in d-galactose-induced aging mice. J. Dairy Sci..

[22] Liu N., Miao S., Qin L. (2020). Screening and application of lactic acid bacteria and yeasts with l-lactic acid-producing and antioxidant capacity in traditional fermented rice acid. Food Sci. Nutr..

[23] Kanno T., Kuda T., An C., Takahashi H., Kimura B. (2012). Radical scavenging capacities of saba-narezushi, Japanese fermented chub mackerel, and its lactic acid bacteria. LWT-Food Sci. Technol..

[24] Liu Y., Ji M., Yu T., Zaugg J., Anesio A.M., Zhang Z., Hu S., Hugenholtz P., Liu K., Liu P. (2022). A genome and gene catalog of glacier microbiomes. Nat. Biotechnol..

[25] Liu C.-F., Tseng K.-C., Chiang S.-S., Lee B.-H., Hsu W.-H., Pan T.-M. (2011). Immunomodulatory and antioxidant potential of Lactobacillus exopolysaccharides. J. Sci. Food Agric..

[26] Lin M.-Y., Yen C.-L. (1999). Antioxidative Ability of Lactic Acid Bacteria. J. Agric. Food Chem..

[27] Amaretti A., Di Nunzio M., Pompei A., Raimondi S., Rossi M., Bordoni A. (2013). Antioxidant properties of potentially probiotic bacteria: In vitro and in vivo activities. Appl. Microbiol. Biot..

[28] Hassan H.M. (1988). Biosynthesis and regulation of superoxide dismutases. Free Radic. Biol. Med..

[29] Yang T., Yang J., Tang K., Zhi W., Chen R., Tan H. (2022). Antioxidative properties analysis of gastrointestinal lactic acid bacteria in Hainan black goat and its effect on the aerobic stability of total mixed ration. Front. Microbiol..

[30] Abdel Tawab F.I., Abd Elkadr M.H., Sultan A.M., Hamed E.O., El-Zayat A.S., Ahmed M.N. (2023). Probiotic potentials of lactic acid bacteria isolated from Egyptian fermented food. Sci. Rep..

[31] Yang J., Dong C., Ren F., Xie Y., Liu H., Zhang H., Jin J. (2021). Lactobacillus paracasei M11-4 isolated from fermented rice demonstrates good antioxidant properties in vitro and in vivo. J. Sci. Food Agric..

[32] Shi S., Dong J., Cheng X., Hu J., Liu Y., He G., Zhang J., Yu H., Jia L., Zhou D. (2022). Biological characteristics and whole-genome analysis of the potential probiotic, *Lactobacillus reuteri* S5. Lett. Appl. Microbiol..

[33] Sun Y., Sun Q., Tang Y., Li Q., Tian C., Sun H. (2023). Integrated microbiology and metabolomic analysis reveal the improvement of rice straw silage quality by inoculation of *Lactobacillus brevis*. Biotechnol. Biofuels Bioprod..

[34] Driehuis F., Oude Elferink S.J.W.H., Van Wikselaar P.G. (2001). Fermentation characteristics and aerobic stability of grass silage inoculated with *Lactobacillus buchneri*, with or without homofermentative lactic acid bacteria. Grass Forage Sci..

[35] Sun L., Xue Y.L., Xiao Y.Z., Te R.G.L., Wu X.G., Na N., Wu N.R., Qili M., Zhao Y., Cai Y.M. (2023). Community Synergy of Lactic Acid Bacteria and Cleaner Fermentation of Oat Silage Prepared with a Multispecies Microbial Inoculant. Microbiol. Spectr..

[36] Li F.H., Ding Z.T., Chen X.Z., Zhang Y.X., Ke W.C., Zhang X., Li Z.Q., Usman S., Guo X.S. (2021). The effects of *Lactobacillus plantarum* with feruloyl esterase-producing ability or high antioxidant activity on the fermentation, chemical composition, and antioxidant status of alfalfa silage. Anim. Feed Sci. Technol..

[37] Yang F., Wang Y., Zhao S., Feng C., Fan X. (2021). Dynamics of the Fermentation Products, Residual Non-structural Carbohydrates, and Bacterial Communities of Wilted and Non-wilted Alfalfa Silage with and Without *Lactobacillus plantarum* Inoculation. Front. Microbiol..

[38] Zhao S., Yang F., Wang Y., Fan X., Feng C., Wang Y. (2021). Dynamics of Fermentation Parameters and Bacterial Community in High-Moisture Alfalfa Silage with or without Lactic Acid Bacteria. Microorganisms.

[39] Liu B., Huan H., Gu H., Xu N., Shen Q., Ding C. (2019). Dynamics of a microbial community during ensiling and upon aerobic exposure in lactic acid bacteria inoculation-treated and untreated barley silages. Bioresour. Technol..

[40] Muck R.E., Nadeau E.M.G., McAllister T.A., Contreras-Govea F.E., Santos M.C., Kung L. (2018). Silage review: Recent advances and future uses of silage additives. J. Dairy Sci..

[41] Borch E., Berg H., Holst O. (1991). Heterolactic fermentation by a homofermentative Lactobacillus sp. during glucose limitation in anaerobic continuous culture with complete cell recycle. J. Appl. Bacteriol..

[42] Isipato M., Dessi P., Sanchez C., Mills S., Ijaz U.Z., Asunis F., Spiga D., De Gioannis G., Mascia M., Collins G. (2020). Propionate Production by Bioelectrochemically-Assisted Lactate Fermentation and Simultaneous CO_2_ Recycling. Front. Microbiol..

[43] Woolford M.K. (1975). The Significance of *Propionibacterium* Species and *Micrococcus lactilyticus* to the Ensiling Process. J. Appl. Bacteriol..

[44] Dong Z., Wang S., Zhao J., Li J., Shao T. (2020). Effects of additives on the fermentation quality, in vitro digestibility and aerobic stability of mulberry (*Morus alba* L.) leaves silage. Asian-Australas. J. Anim. Sci..

[45] Filya I., Muck R.E., Contreras-Govea F.E. (2007). Inoculant effects on alfalfa silage: Fermentation products and nutritive value. J. Dairy Sci..

[46] Rafińska K., Pomastowski P., Wrona O., Górecki R., Buszewski B. (2017). Medicago sativa as a source of secondary metabolites for agriculture and pharmaceutical industry. Phytochem. Lett..

[47] Zhang Y., Usman S., Li Q., Li F., Zhang X., Nussio L.G., Guo X. (2024). Effects of antioxidant-rich *Lactiplantibacillus plantarum* inoculated alfalfa silage on rumen fermentation, antioxidant and immunity status, and mammary gland gene expression in dairy goats. J. Anim. Sci. Biotechnol..

[48] Tian X.Z., Paengkoum P., Paengkoum S., Chumpawadee S., Ban C., Thongpea S. (2019). Short communication: Purple corn (*Zea mays* L.) stover silage with abundant anthocyanins transferring anthocyanin composition to the milk and increasing antioxidant status of lactating dairy goats. J. Dairy Sci..

[49] Kachouri F., Ksontini H., Kraiem M., Setti K., Mechmeche M., Hamdi M. (2015). Involvement of antioxidant activity of *Lactobacillus plantarum* on functional properties of olive phenolic compounds. J. Food Sci. Technol..

[50] Pilarczyk B., Jankowiak D., Tomza-Marciniak A., Pilarczyk R., Sablik P., Drozd R., Tylkowska A., Skolmowska M. (2012). Selenium concentration and glutathione peroxidase (GSH-Px) activity in serum of cows at different stages of lactation. Biol. Trace Elem. Res..

[51] Yang F., Chen C., Ni D., Yang Y., Tian J., Li Y., Chen S., Ye X., Wang L. (2023). Effects of Fermentation on Bioactivity and the Composition of Polyphenols Contained in Polyphenol-Rich Foods: A Review. Foods.

[52] Liu W., Cui X., Zhong Y., Ma R., Liu B., Xia Y. (2023). Phenolic metabolites as therapeutic in inflammation and neoplasms: Molecular pathways explaining their efficacy. Pharmacol. Res..

[53] Dong X., Qi J., Xu K., Li B., Xu H., Tian X., Lei H. (2023). Effect of lactic acid fermentation and in vitro digestion on the bioactive compounds in Chinese wolfberry (*Lycium barbarum*) pulp. Food Biosci..

[54] Isas A.S., Escobar F., Álvarez-Villamil E., Molina V., Mateos R., Lizarraga E., Mozzi F., Van Nieuwenhove C. (2023). Fermentation of pomegranate juice by lactic acid bacteria and its biological effect on mice fed a high-fat diet. Food Biosci..

[55] Mohd Zin Z., Azman S.N.S., Yahya F., Hasmadi M., Zainol M.K. (2022). Effect of pH and temperature on antioxidant enzymes activities in *Morinda citrifolia* L. (Mengkudu) leaves extract. Food Res..

[56] Aguirre J.D., Culotta V.C. (2012). Battles with iron: Manganese in oxidative stress protection. J. Biol. Chem..

[57] Archibald F.S., Duong M.N. (1984). Manganese acquisition by *Lactobacillus plantarum*. J. Bacteriol..

[58] Prabhakar R., Vreven T., Morokuma K., Musaev D.G. (2005). Elucidation of the mechanism of selenoprotein glutathione peroxidase (GPx)-catalyzed hydrogen peroxide reduction by two glutathione molecules: A density functional study. Biochemistry.

